# A Study on Evaluating Cardiovascular Diseases Using PPG Signals

**DOI:** 10.3390/bioengineering12121283

**Published:** 2025-11-21

**Authors:** Lei Wang, Meng-Yu Hsiao, Zi-Jun Chen, Ruo-Jhen Wu, Meng-Ting Wu

**Affiliations:** 1 Department of Electrical Engineering, Feng Chia University, Taichung 40724, Taiwan; leiwang@fcu.edu.tw (L.W.); d1019560@o365.fcu.edu.tw (M.-Y.H.); d1055690@o365.fcu.edu.tw (Z.-J.C.); d1055656@o365.fcu.edu.tw (R.-J.W.); 2 Division of Neurosurgery, Department of Surgery, Cheng-Hsin General Hospital, Taipei 11220, Taiwan; 3 Department of Neurological Surgery, Tri-Service General Hospital, National Defense Medical Center, Taipei 11490, Taiwan; 4 Ph.D. Program of Electrical and Communications Engineering, Feng Chia University, Taichung 40724, Taiwan

**Keywords:** photoplethysmography, arteriosclerosis, arterial stiffness, cardiovascular disease

## Abstract

The widely used oximeter design was adopted and improved as the configuration mainframe in this study to acquire PPG signals. When users wear a finger probe and input their height, the device acquires PPG signals through the probe circuit, then filters and amplifies the signals to remove unnecessary noise, and uses an ARM-M4 to analyze the main peak, dicrotic wave, and wave valley of the PPG waveform to calculate related indexes for the final assessment. After 100 s, the HRV, sine wave ratio, and SI results are estimated, and a cardiovascular disease risk assessment is presented using a risk level from 0 to 5. This study uses the stiffness index (SI), sine wave ratio (SIN), and heart rate variability (HRV) to assess cardiovascular status. The SI is derived from PPG signal characteristics and reflects vascular stiffness based on blood flow rebound time. However, some PPG signals lack a dicrotic wave (sine waves), which is often caused by severe arterial stiffness. These waveforms lead to errors in SI calculation due to misidentification of the dicrotic wave. The appearance of a sine wave indicates that blood pulsation is abnormal; however, it will make the SI calculation algorithm produce a seemingly normal health performance. To address this, the auxiliary line method was introduced to identify sine waves, and the SIN ratio occurring in contiguous PPG waves was incorporated to calculate their proportion in PPG signals, aiding SI analysis and arterial stiffness evaluation. The total power (TP) value obtained via HRV frequency-domain analysis reflects autonomic nervous activity. As reduced autonomic function may relate to cardiovascular diseases, TP is included as an evaluation indicator. By analyzing PPG signals, calculating SI and SIN, and integrating the HRV indicator, this study evaluates arterial stiffness and cardiovascular health, helping participants understand their physical condition more quickly and conveniently, and potentially preventing cardiovascular diseases at an early stage.

## 1. Introduction

According to the World Health Organization, cardiovascular disease remains one of the leading causes of mortality worldwide. In Taiwan, statistics from the Ministry of Health and Welfare identify cardiovascular disease as the second leading cause of death [1]. Major risk factors include physical inactivity, unhealthy diet leading to hypertension, hyperlipidemia, and hyperglycemia, as well as smoking and excessive alcohol consumption. Yet approximately 80% of acute cardiac events, such as myocardial infarction and stroke, are preventable through timely intervention. The majority of cardiovascular disease originates from vascular plaques of fat and metabolic waste, and the location of vascular deposits affects the severity of cardiovascular disease [2]. Arterial stiffness is therefore regarded as a direct marker of cardiovascular risk. By evaluating relevant biomarkers or applying vascular imaging techniques, clinicians can monitor vascular flow and circulation to assess the likelihood of cardiovascular disease at the time of measurement.

Currently, hospital-based assessments of arterial stiffness are performed using either invasive or non-invasive methods. Invasive procedures include coronary angiography [3] and contrast-enhanced coronary angiography [4], both of which require injection of contrast agents and medications. Non-invasive assessments include ankle–brachial index (ABI) [5] and pulse wave velocity (PWV) [6]. However, these approaches are restricted to the hospital setting due to requirements of specialized medical equipment and trained personnel, making them unsuitable for routine home monitoring.

In recent years, wearable devices such as smart wristbands have become widely available, providing modern, convenient, and painless health monitoring through optical sensing technology [7]. To enable simple, home-based assessment of arterial stiffness, non-invasive photoplethysmography (PPG) offers a promising approach.

PPG is an optical measurement technique in which LED light is projected onto the skin, with the light absorbed or transmitted through underlying tissue detected by probes and converted into physiological signals. These signals provide information on blood flow and allow estimation of a variety of physiological parameters [8]. This technique is non-invasive and can be applied to a broad range of medical devices. The pulse oximeter is a familiar example of PPG technology. Other applications include the measurement of blood pressure [9], blood glucose [10], heart rate [11], respiration [12], and arterial stiffness [13]. Compared with conventional diagnostic methods, PPG is non-invasive and highly accessible.

In this study, we developed an easy-to-use device for evaluating cardiovascular health status with a system consisting of a fingertip detector and an LCD touchscreen for inputting the subject’s height. After a brief 100 s measurement, the device outputs heart rate variability (HRV), sine wave ratio, and stiffness index (SI) on the LCD screen. Based on these measurement results, the subjects’ cardiovascular health status and risk level for cardiovascular disease will be assessed and shown by the device.

The structure of this study is arranged as follows. Section 2 focuses on the system design for acquiring PPG waveforms and introduces the methods for calculating SI and HRV indexes. Section 3 presents the major cardiovascular health indexes (SI and HRV) used in this study and describes their correlations with coronary artery disease (CAD). This section also explains that inherent errors in identifying the dicrotic peak may reduce the accuracy of certain SI values; therefore, this study proposes an improvement and introduces a third index (sine wave ratio). Section 4 presents the measurement results obtained from 191 participants of different age groups. Section 5 proposes a cardiovascular status assessment framework based on the aforementioned measurements and analysis results. Section 6 concludes the study.

## 2. System Design

The commonly used oximeter architecture was adopted and improved as the main configuration framework in this study in order to acquire non-distorted PPG signals while considering human breathing frequency for determining filtering and sampling settings. This ensures that the acquired PPG signals meet the calculation requirements of SI and HRV indexes and further enables more accurate SI and HRV extraction through specific algorithms.

**Figure 1:**
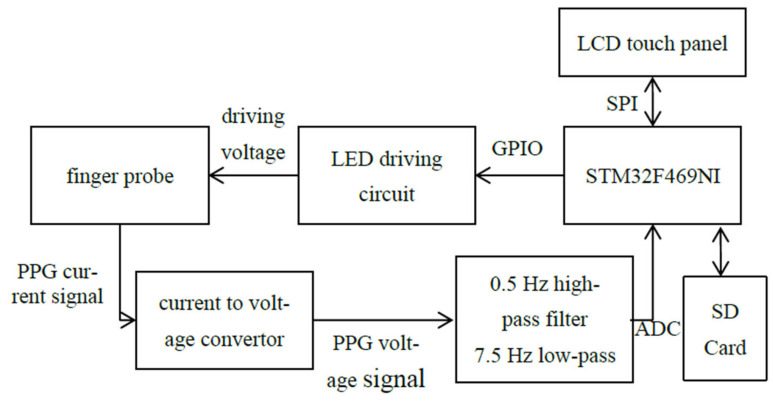
System configuration.

The system configuration used to acquire PPG signals is shown in Figure 1. Due to the relatively complex mathematical computations required in this study, the STM32F469NI ARM-M4 microprocessor was selected to meet the computational demands. Subjects first input their height using the LCD touch panel and then wear the finger probe. The red LED on the probe provides illumination that penetrates the subject’s finger, and the photodetector receives the transmitted light and generates current signals. These current signals are converted into voltage signals, filtered, and amplified by the hardware circuitry to obtain the AC component of the PPG waveform. The microprocessor digitizes the waveform through the ADC interface and completes PPG sampling and SI/HRV calculation within 100 s. The resulting biological information, including HRV total power (TP) and SI, is displayed on the LCD panel through the SPI interface.

**Figure 2:**
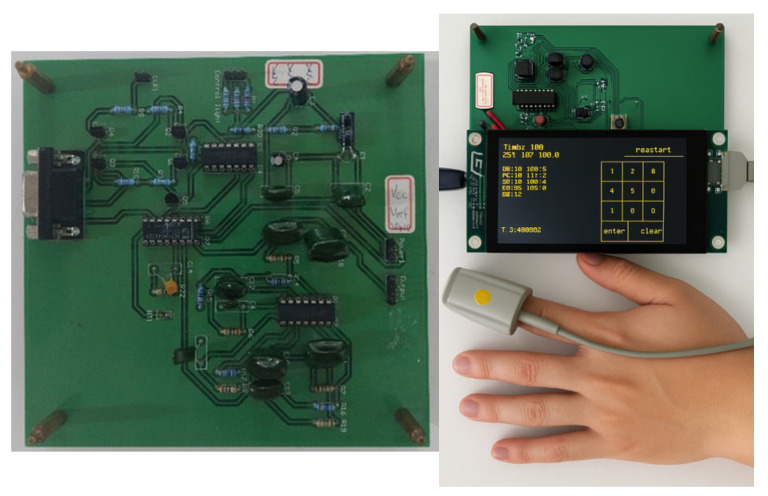
Illustration of completed circuit and detection instrument.

Regarding the probe driving and signal sampling circuit, a BJT was adopted as the LED driving component to ensure stable illumination for the red-light LED on the probe. The signal received by the photodetector is a current signal; therefore, a current-to-voltage conversion circuit is required so that the microprocessor can acquire the PPG waveform through the ADC interface. Since excessive noise may cause misjudgment during waveform analysis, a high-pass filter with a cutoff frequency of 0.5 Hz is used to remove breathing signals and DC components, while a low-pass filter with a cutoff frequency of 7.5 Hz is used to suppress electrical noise and amplify the PPG signal for further processing by the microprocessor. In addition, an SD card is included to store the PPG signals for further analysis. The complete circuit and instrument design are shown in Figure 2.

**Figure 3:**
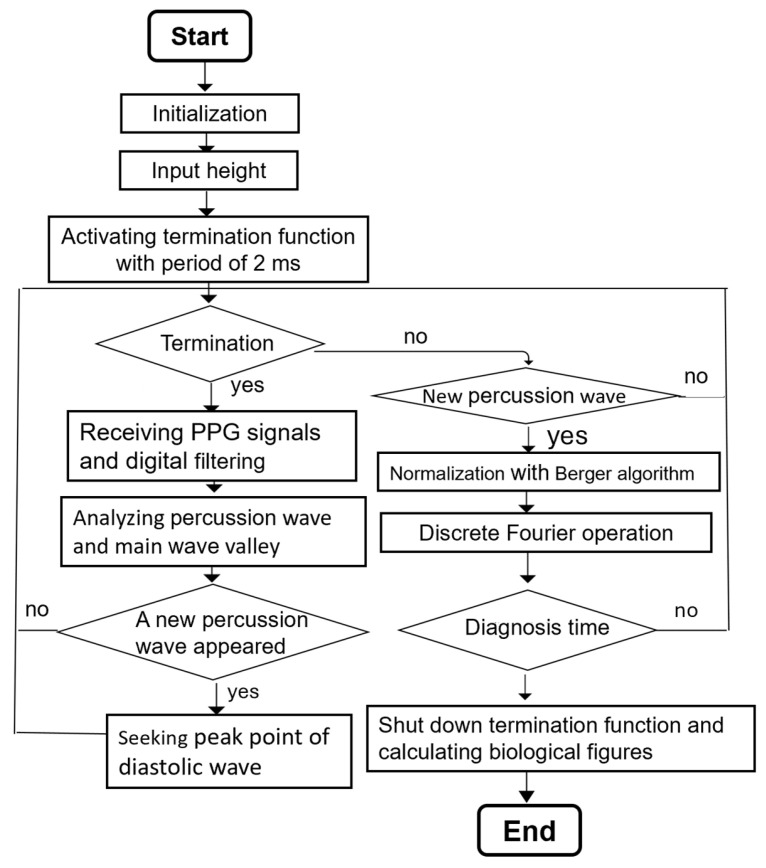
The flowchart of the firmware.

The main flowchart of the system program is shown in Figure 3. It first conducts initialization to complete the set-up process of the interfaces, then waits for the user to input their height through the LCD touch panel. After the initial settings are completed, the termination function of the microprocessor will set to terminate once at 500 Hz. When it is terminated, the PPG signal is received through the ADC interface and filtered and amplified by the circuit, and the 60 Hz noise is deleted by the digital filter so that a smooth PPG waveform is acquired. Further assessment may determine whether the PPG waveform contains percussion waves and wave valleys, in order to confirm the existence of the completed PPG signal and judge the peak point of the diastolic wave from said completed PPG signal. As for the main program, the Berger algorithm [14] is operated when discovering a new percussion wave, so that the intervals of heartbeats are normalized. Furthermore, discrete Fourier transformation is conducted on those heartbeat intervals after normalization in order to acquire HRV values. The overall calculation time is 100 s, after which the functions and main program are terminated and the average is taken for all SI indexes collected. The diagnosis results are displayed on the LCD touch panel through the SPI interface.

Although the original PPG signals are processed by the filter circuit to delete noise lower than 0.5 Hz and above 7.5 Hz, high-frequency noise may still exist. It is necessary to filter out this type of noise by adopting a digital filter. Hence, this study applies a finite impulse response filter to delete noise, setting the number of layers N as 41 and the cut-off frequency f_c_ as 7.5 Hz. The sampling frequency of the microprocessor f_s_ is set as 500 Hz. The normalization frequency ω_cn_ should be 0.03π, according to Equation (1).

(1) $$\omega_{cn}=2\cdot \pi \cdot \frac{f_{c}}{f_{s}}$$

By adopting the normalization Sinc function shown in Equation (2), we may obtain the ideal pulse response sequence h(n).

(2) $$h\left(n\right)=\left\{\begin{array}{c} \frac{\sin\left(\omega_{cn}\cdot n\right)}{\pi \cdot n},\;|n|\leq \frac{N-1}{2}\\ \;\;\;\omega_{cn\;},\;n=0 \end{array}\right.$$

The window functions are adopted to enhance filter effectiveness. And the Hamming window of the window functions may have a relatively narrower transition band, thus acquiring better filtering effectiveness. We therefore adopt the Hamming window to conduct filter function and use Equation (3) to present the Hamming window sequence.

(3) $$\omega \left(n\right)=\left\{\begin{array}{c} 0.54-0.46\cdot \cos\left(\frac{2\pi n}{N-1}\right),\;|n|\leq \frac{N-1}{2}\\ 0\;\;\;\;\;\;\;\;\;\;\;\;\;\;\;\;\;\;\;\;\;\;\;\;\;\;\;\;\;\;\;\;\;\;\;\;,\;otherwise \end{array}\right.$$

As *n* shifts from 0 to *N* − 1, and the obtained Sinc function is adopted, the window function sequence applies Equation (4) to allow the original PPG signal *x*(*k*) to conduct the digital filter process in order to transfer into a relatively smooth signal *y*(*k*).

(4) $$y\left(k\right)=\sum_{n=0}^{N-1}x(k+n)\cdot h(n)\cdot \omega \left(n\right)$$

## 3. Arterial Stiffness Indicators

**Figure 4:**
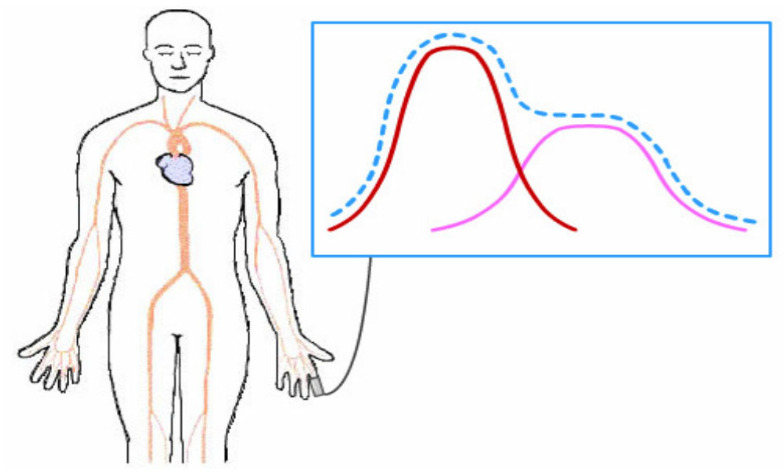
Schematic diagram of a PPG signal [15].

This study utilizes PPG signals derived from light transmitted through blood vessels, from which information on vascular diameter can be obtained. Because vascular diameter changes with the heart’s output, the PPG signal carries encrypted information about the vascular system. Figure 4 illustrates a typical, complete PPG waveform.

When the heart contracts and ejects blood, the diameter of blood vessels changes. As the pressure wave reaches the measurement site, the first prominent peak, i.e., the primary wave peak, appears. A reflected pressure that appears as a second wave, known as the dicrotic wave, follows as the pulse travels to the periphery vasculature [16]. A complete PPG waveform therefore consists of a primary wave peak and a dicrotic wave, as shown by the dashed line in Figure 4.

### 3.1. SI Value Calculation

This study first follows the method of Millasseau and colleagues [17], who proposed the SI to quantify the relationship between the primary wave peak and the dicrotic wave as an indicator of arterial stiffness.

The SI is calculated using Equation (5) shown below, where ∆T (s) is the time interval between the primary and reflected pulse and h (m) is the subject’s height. A higher SI value indicates a faster propagation of the dicrotic wave, signifying greater vascular stiffness. On the contrary, a lower SI value reflects a slower transmission of the dicrotic wave and better vascular elasticity.

SI = h/∆T(5)

**Figure 5:**
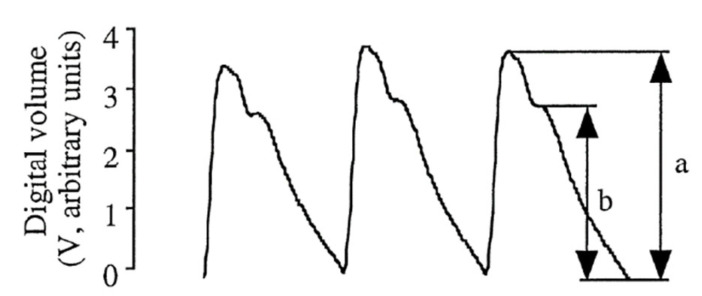
Schematic diagram of RI [17].

The reflection index (RI) derived from PPG signals can also provide information on arterial stiffness [18]. As shown in Figure 5 and Equation (6), RI is the amplitude ratio of the dicrotic wave to the primary wave.

(6) $$RI=\frac{b}{a}\times 100\%$$

**Figure 6:**
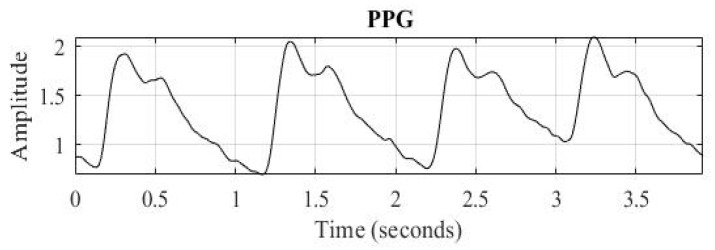
Schematic diagram of PPG signal.

Greater vascular elasticity results in less amplitude attenuation of the dicrotic wave and hence a higher RI value; reduced elasticity leads to greater attenuation of the dicrotic wave and a lower RI value. However, the RI is less reliable than the SI as an indicator due to greater overlap of the two waves when the time interval between the two waves shortens, resulting in a misleading increase in the amplitude and the RI. Figure 6 shows a real example from a subject with arterial stiffness, whose ∆T was 0.232 s and SI 7.32, indicating a high arterial stiffness, but with an remarkably high RI of 87.8% that falsely suggests good vascular elasticity. Thus, the RI is not suitable for measuring arterial stiffness despite being useful for observing changes in vascular blood flow. This study therefore uses the SI to evaluate arterial stiffness.

**Figure 7:**
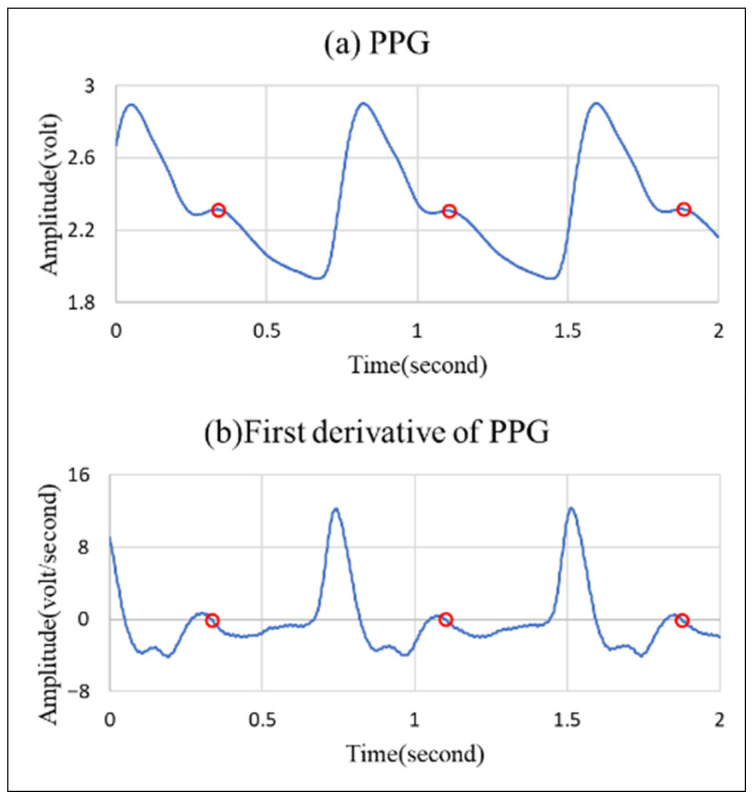
(**a**) Illustration of a normal PPG signal. (**b**) Illustration of the first derivative of a normal PPG signal.

When calculating SI values in normal practice, the PPG signal is first differentiated once to locate the dicrotic wave, which lies between the peak and trough of the primary wave. The point at or closest to zero within this time interval is identified as the dicrotic wave, as illustrated by Figure 7. SI is then determined using Equation (5).

**Figure 8:**
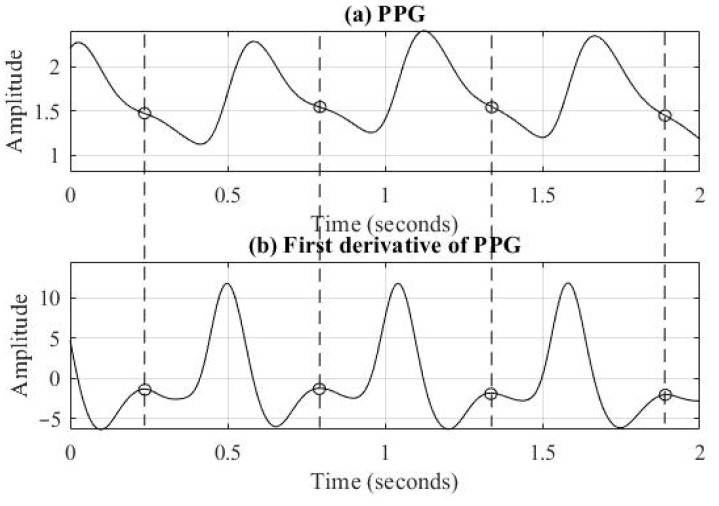
(**a**) Unapparent diastolic wave PPG signal; (**b**) the first derivative diagram of this signal.

As a diastolic wave is unapparent, it is necessary to seek the point with the first derivative value closest to zero between the percussion wave and the valley. This point would be defined as the diastolic wave peak point. As indicated by Figure 8b, only the main percussion wave and the main wave valley may pass through the zero point; therefore, we can only treat the closest point to zero as the diastolic wave peak point, as indicated by o in the figure. This corresponds to Figure 8a, showing the peak point of the unapparent diastolic wave.

During preliminary experiments, while expecting but not observing a high SI value in patients with diseases related to arterial stiffness, we found low SI values in many elderly subjects and in patients with known cardiovascular disease. This SI pattern misaligned with the anticipated age-related effects [17,19], suggesting errors in SI estimation.

### 3.2. Definition of Sine Waveform and SIN Wave Ratio

**Figure 9:**
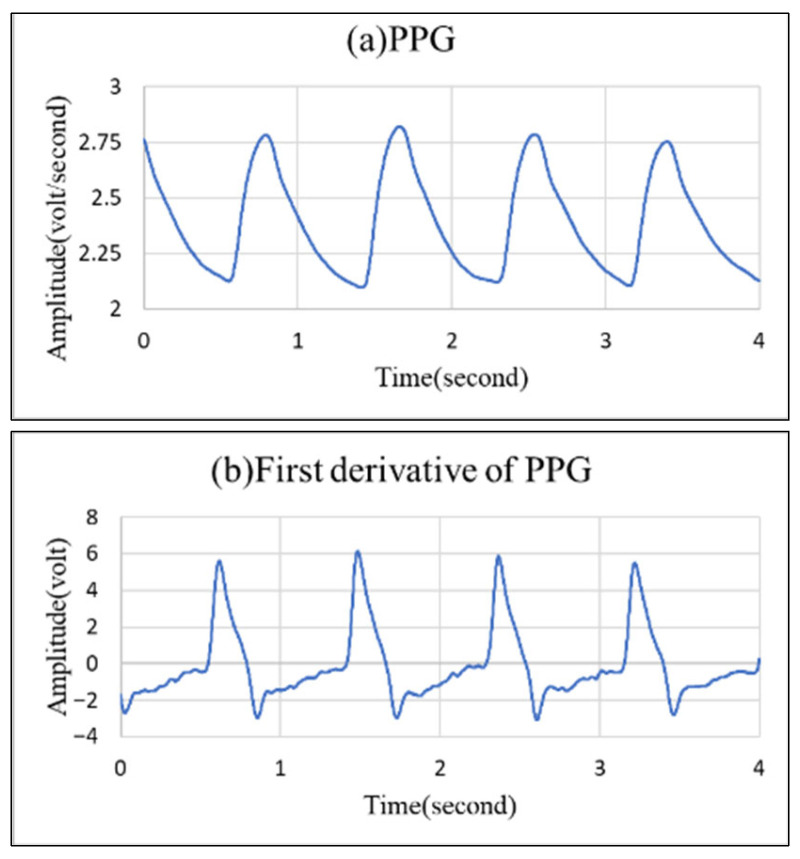
(**a**) Sine waveform illustration of PPG signal. (**b**) First derivative illustration of sine waveform in PPG signal.

To investigate the cause of inaccuracies in SI estimation, PPG signals were retested from suspected subjects using an oscilloscope. The signals were frequently found to exhibit the characteristic pattern shown in Figure 9a. This specific pattern, termed the “sine wave” (SIN) [20], is notable for the absence of a dicrotic wave. Thus, SI calculation using the first derivative method would provide a falsely low value, as shown in Figure 9b. The loss of the dicrotic wave can be attributed to vascular wall stiffening, luminal narrowing, and loss of elasticity as a result of vascular aging or accumulation of metabolic waste and lipid, dampening the second wave before it reaches the measurement site [21]. Moreover, the pulse energy caused by the heartbeat may also affect the scale of diastolic wave energy. We therefore may assess the conditions of vascular vessels and blood circulation situations and help to judge the effect of the associated disease based on whether PPG signals of the subjects show sine waves.

**Figure 10:**
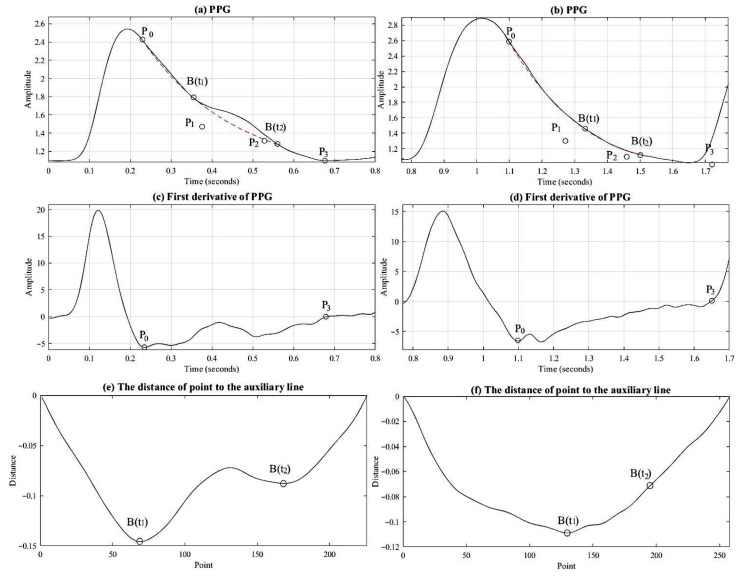
(**a**) PPG containing diastolic wave; (**b**) PPG of the sine wave; (**c**) first derivative of (**a**); (**d**) first derivative of (**b**); (**e**) calculating distance from all points to auxiliary line (**a**); (**f**) calculating distance from all points to auxiliary line (**b**).

The study adopted the relatively easy and efficient Bézier curve scheme to draw a smoother curve [22], indicating a sine wave. This drawing scheme may be divided into the following two types:

1. As there are two or more valley points from all points to the auxiliary line, as indicated by Figure 10a, we take the two points, the one nearest to the percussion wave *B*(*t*_1_) and the one nearest to the wave valley B(t_2_), that expressed the existing diastolic wave in this designated range. Then, we adopt the third-order Bézier curve equation in Equations (7) and (8) in order to calculate two unknown control points, *P*_1_ and *P*_2._ Among them, *P*_0_ and *P*_3_ are the first lowest point of the first derivative and the valley point of the PPG signals, as indicated by Figure 10c. Also, to reduce the range of search, we only draw out from *P*_0_ to the sine wave, as indicated by the dotted line marked in Figure 10a.
  (7) $$t=\frac{B\left(t\right)-P_{0}}{P_{3}-P_{0}},0\leq t\leq 1$$
  (8) $$B\left(t\right)=P_{0}{\left(1-t\right)}^{3}+3P_{1}{\left(1-t\right)}^{2}t+3P_{2}\left(1-t\right)t^{2}+P_{3}t^{3}$$
2. As indicated by Figure 10f, when there is only one valley in the distance from all points to auxiliary B(t_1_), it is implied that it is an unapparent dicrotic notch or an unapparent diastolic wave. We may adopt Equation (9) to calculate integral *B*(*t*_2_). Subsequently, Equation (8) was adopted to determine control points *P*_1_ and *P*_2_, as well as drawing the sine curve from *P*_0_ to *B*(*t*_2_), as indicated by the dotted line in Figure 10b.
  (9) $${B(t}_{2})=\left\lfloor \frac{P_{3}-B(t_{1})}{2}\right\rfloor$$

**Figure 11:**
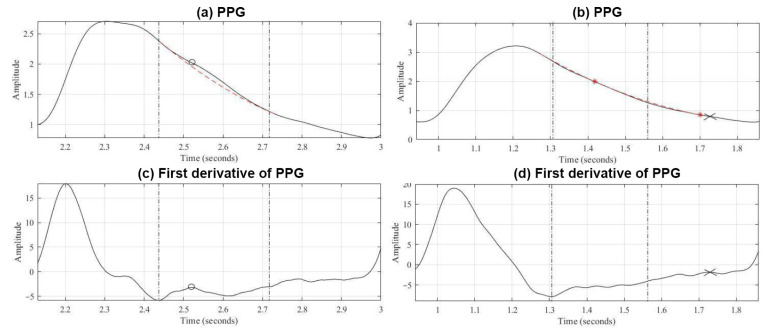
(**a**) PPG containing diastolic wave; (**b**) PPG of the sine wave; (**c**) first derivative of (**a**); (**d**) first derivative of (**b**).

Furthermore, based on the correlation Equation (10), we acquired the correlation between the unapparent dicrotic notch curve drawn by the Bézier curve and the PPG waveforms. A lower correlation would indicate that the possibility of the PPG signal containing a diastolic wave was higher. As shown by Figure 11a, the correlation coefficient is 99.49%. The dotted line interval is the Bézier curve. We can also find locations higher than the Bézier curve in the range of the diastolic wave. From this range and based on the first derivative scheme, we can find the correct peak point of the diastolic wave, as in Figure 11a. If we only apply the first derivative scheme, then we find point x in Figure 11c, which may lead to a misjudgment.

(10) $$r=\frac{n\sum xy-\sum x\sum y}{\sqrt{n\sum x^{2}-{\left(\sum x\right)}^{2}}\sqrt{n\sum y^{2}-{\left(\sum y\right)}^{2}}}$$

Figure 11b is the sine wave. Its waveform almost overlaps with the Bézier curve, and the correlation coefficient is as high as 99.96%. If we only apply the first derivative scheme to analyze the wave peak of the diastolic wave, then we may find a range other than the Bézier curve, as indicated by point x in Figure 11d. Hence, we may adopt the Bézier curve to precisely identify whether the PPG waveform can be the sine wave.

In order to determine whether it is sine wave, we need to set a threshold for the correlation coefficient. We therefore randomly selected 50 waveforms from the subjects and put them in order according to the correlation coefficient, and referred to the oscillatoscope for the waveforms to judge whether they were the sine wave. Among the 50 waveforms collected, 32 waveforms exhibited obvious normal PPG waves with correlation coefficients ranging from 92.85% to 99.83%, and 18 waveforms exhibited obvious sine waves with correlation coefficients ranging from 99.86% to 99.99%. Based on this observation, the correlation coefficient threshold was set to 99.85%. If the correlation coefficient was greater than this threshold, it was considered to be a sine wave.

In order to confirm the precision of the improved scheme for this study, we conducted another experiment in the hospital and used the oscillatoscope to verify the correctness of the improved scheme. The whole process was compared with the first derivative scheme. There were three subjects with confirmed CAD and another 26 subjects without a confirmed diagnosis of CAD.

The misjudged SI in the experiment was zero, thus indicating the excellent effectiveness of the improved scheme. Furthermore, we noticed that there were 11 subjects with a sine wave ratio of more than 60%. Seven of the eleven subjects had confirmed cardiovascular disease affecting their health. One of the subjects had motion problems in both feet and the other three were aged above 50. We therefore noticed that with a high ratio of sine waves, the probability of having cardiovascular disease or diabetes is very high. In addition, as arterial stiffness becomes more serious with age, we can judge the vascular vessel situation of subjects by sine wave ratio.

### 3.3. HRV Total Power

During preliminary trials, we found that physical states could influence both SI and SIN, such as extreme fatigue, which produced abnormal results. To minimize such confounding factors, items regarding fatigue, i.e., working for more than 15 h or experiencing insomnia, were included in the questionnaire administered to the subjects prior to evaluation.

HRV, which reflects the autonomous nervous activity, was included as an additional component in the present study. Reduced autonomous nervous function is associated with conditions such as cardiovascular disease and diabetes [23], and it can also be transiently affected by physical and psychological stress. Thus, HRV provides valuable insight into cardiovascular risk. If a subject reports no fatigue yet exhibits abnormal HRV, an underlying medical condition affecting their physiological performance may be suspected.

**Figure 12:**
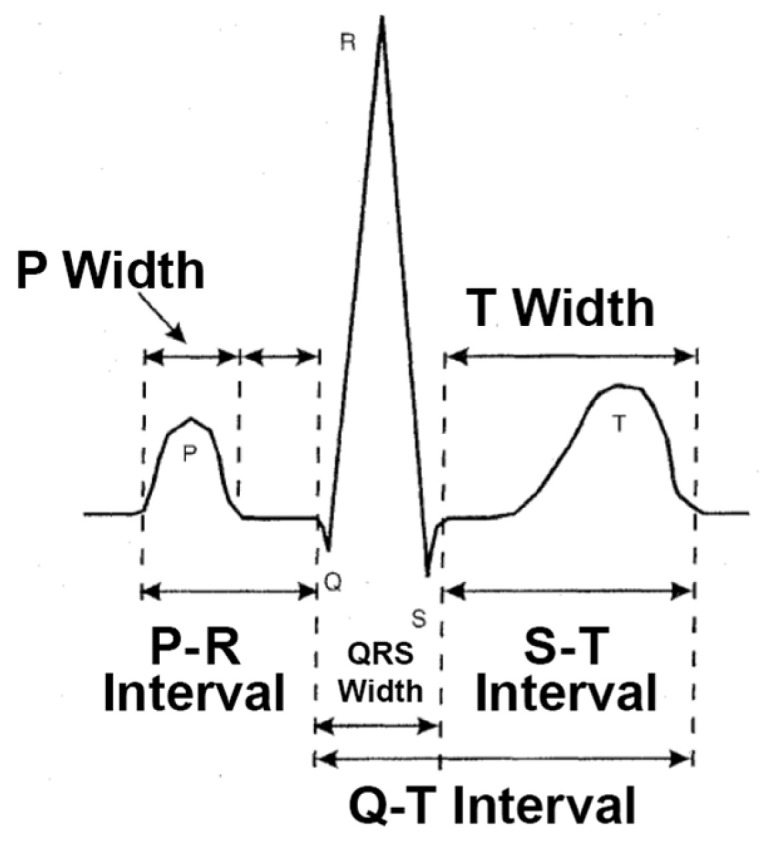
Schematic figure of PQRS waves on ECG [25].

HRV is assessed by electrocardiography (ECG), which detects myocardial depolarization through surface electrodes. The voltage differences during depolarization are plotted on ECG recording paper, producing specific waveform characteristics (Figure 12). These were described and defined as P, QRS, and T waves by Einthoven and colleagues in 1903 [24]. HRV is calculated from RR intervals (RRIs) to assess the cardiovascular condition of evaluated subjects.

**Figure 13:**
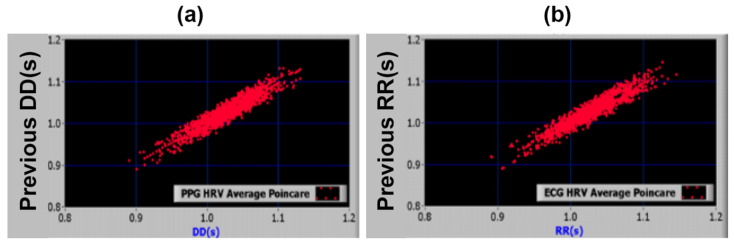
Poincaré diagrams showing HRV derived from PPG (**a**) and ECG (**b**) [26].

Bolanos et al. (2006) found a >97% correlation between RR intervals derived from ECG and the DD intervals from PPG [26] (Figure 13). Because ECG requires multiple sensing points while PPG needs only one, PPG was chosen over ECG for convenience to assess the subjects’ HRV in the present study.

HRV can be analyzed using time-domain and frequency-domain indices. The time-domain index reflects the dispersion of heartbeat intervals based on long-term statistics.

In contrast, the frequency-domain index is derived from normalized, short-term heartbeat intervals using discrete Fourier transformation to obtain the power spectrum, which is then divided into three principal segments and forms the basis of HRV assessment [27].

Total power (TP) represents the power spectrum from 0 to 0.4 Hz and is clinically meaningful for an overall assessment of HRV.

High-frequency power (HFP) represents the spectrum from 0.15 to 0.4 Hz and is clinically meaningful for the assessment of parasympathetic nervous activity.

Low-frequency power (LFP) represents the spectrum from 0.04 to 0.15 Hz and is clinically meaningful for the assessment of sympathetic nervous activity.

Very low-frequency power (VLFP) represents the spectrum below 0.04 Hz. Its clinical meaning has not yet been established by research.

Because the time-domain analysis commonly requires 24 h of data, we selected the frequency index to allow for rapid HRV assessment. Furthermore, TP was chosen because it reflects the subjects’ overall physical and psychological state and diminishes confounding from transient external factors such as physical fatigue or sleep deprivation, preventing misinterpretations unrelated to disease. Moreover, cross-examination using questionnaire responses and medical charts could verify low TP related to cardiovascular disease.

**Figure 14:**
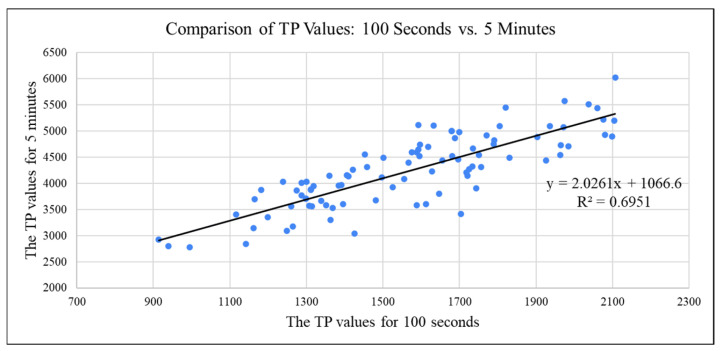
TP values measured over 5 m and 100 s.

**Figure 15:**
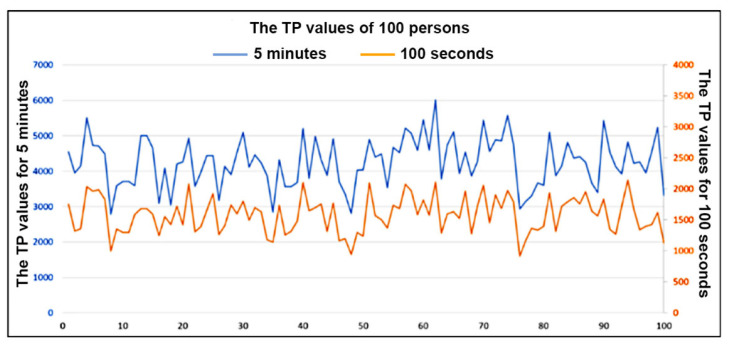
Difference between two TP results for the 100 patients.

Measurement of heart rate variability (HRV) by frequency-domain indices requires discrete Fourier transformation. However, because heartbeats are not perfectly regular, the time-domain index cannot use the same sampling period to calculate the intervals between heartbeats. Therefore, we applied the algorithm of Berger et al. (1986) [14], which normalizes data at exactly 4 Hz and converts the heartbeat interval sequence by discrete Fourier transformation into power spectrum density (PSD), from which TP is derived. Continuous data of at least 5 min is recommended for reliable frequency-domain HRV assessment [28]. Considering the subjects’ broad age range and the possibility of signal interruption due to hand tremors, 100 s signals was used to ensure data continuity in the present study. This approach facilitates rapid signal acquisition and reduces technical difficulties in obtaining stable waveforms. Unstable signals were filtered by the device’s integrated circuit during data collection. To validate the accuracy of the approach, we compared HRV results from 100 s and 5 min data in 100 subjects from the MIMIC database [29,30]. As shown in Figure 14, TP values derived from 5 min and 100 s recordings demonstrated similar trends. Plotting each subject’s TP values from both timeframes yielded a correlation coefficient (R) of 0.8337, indicating a strong linear association between the two datasets. As shown in Figure 15, although a consistent offset was noted between the two sets of TP values, this could be corrected by a slight adjustment of the normal TP range for 100 s data.

## 4. Preliminary Analysis Results

This study utilized 100 s PPG signals from 191 subjects, who were recruited between 2022 and 2024 from the outpatient clinics and management staff of Cheng Hsing General Hospital, as well as the administrative department and students of Feng Chia University. Individuals with arrythmia or implanted pacemakers were excluded. This study was approved by the Institutional Review Board (No. (770) 109-10), and all subjects provided written consent. All procedures complied with the 1975 Declaration of Helsinki.

### 4.1. Observation of SIN Ratio

**Figure 16:**
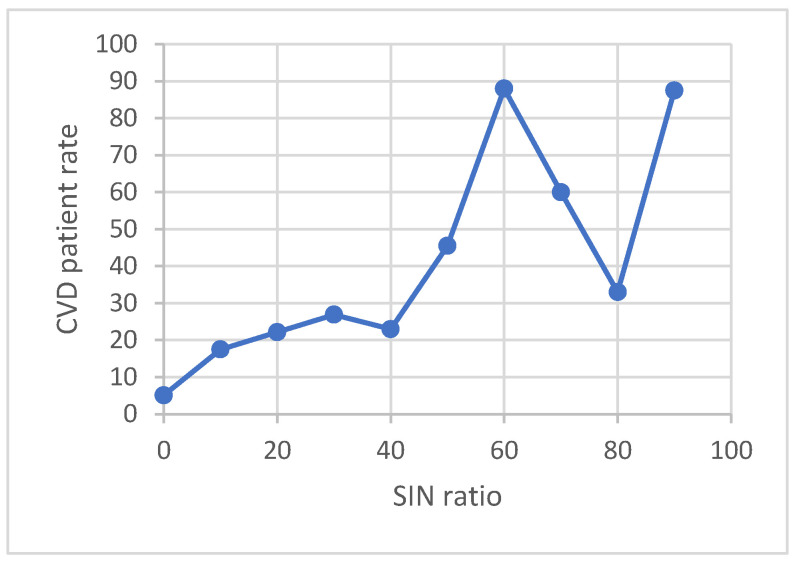
CVD patient rate over various SIN ratios.

As shown in Figure 16, once the SIN ratio exceeds 40%, the proportion of subjects with CVD increases markedly. Therefore, in this study, an abnormal SIN value was defined as >40% based on the observed distribution of the collected measurements. The proportion of subjects with abnormal SIN was calculated and expressed as a percentage of the entire study cohort (Table 1). An age-related rise in the proportion of subjects with abnormal SIN was observed, consistent with the expectation that vascular elasticity declines with age. When subjects were stratified by the presence of known cardiovascular disease, almost half (43%) of those with cardiovascular disease had a SIN >40%, compared to only 13% of those without. This clear difference suggests that most of the subjects with known cardiovascular disease had reduced vascular elasticity due to poor cardiovascular health. Nevertheless, the difference between subgroups was less prominent in subjects aged 60 years and above, implying that high SIN may occur in some elderly individuals regardless of known cardiovascular disease.

By setting the abnormal SIN threshold at 40%, 58% (25/(25 + 18)) of subjects with abnormal SIN had known cardiovascular disease. When focusing on individuals with SIN >80%, six of seven (87.5%) had known cardiovascular disease, while the remaining subject was underweight and had a history of severe comorbidities of kidney and liver cysts, despite not having known cardiovascular disease. This indicates that serious non-cardiovascular illnesses may also produce abundant sine waves. Based on these results, a two-staged threshold could help the accuracy of SIN abnormality determination.

### 4.2. Observation of SI

**Figure 17:**
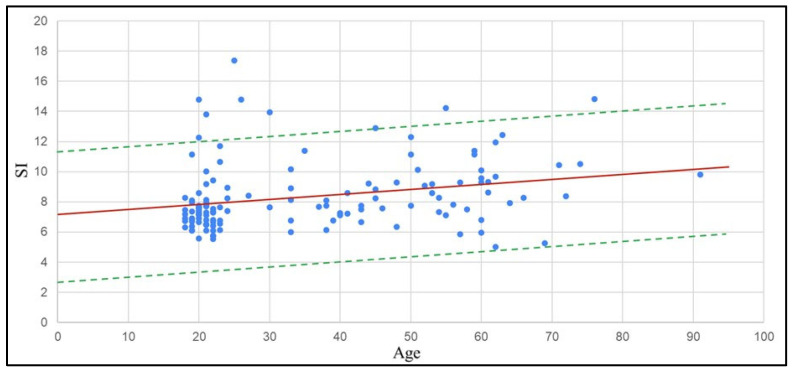
Scatter plot of SI values for subjects without cardiovascular disease.

The mean SI value among subjects with known cardiovascular disease (9.09 m/s) was found to be higher than the 8.32 m/s observed in those without, illustrating increased arterial stiffness in cardiovascular patients. Hence, data from the 57 subjects with known cardiovascular disease was excluded from estimating the SI normal reference range to reduce confounding. Following a previously published method [18], SI results from the remaining 134 subjects, who had no known cardiovascular disease, were regressed against age, as shown by the red line in Figure 16. The dashed lines represent the 95% confidence interval, and the area between the two dashed lines defines the age-specific normal reference range for the SI. Table 2 lists the reference values in the 5-year age interval. From Figure 17, data points were found to cluster among adolescents and young adults, due to all cases of diagnosed cardiovascular disease in subjects aged above 30. The proportion of subjects with confirmed cardiovascular disease also increased with age. As subjects with known cardiovascular disease were excluded, results distributed in the figure become unbalanced according to age distribution, with fewer older participants.

Table 3 shows the proportion of subjects with elevated SI values across age groups. The proportion of subjects with abnormal SI values increased with age, implying a possible correlation between age and SI, where the likelihood of abnormal SI values increases with age. In the present study, subjects with known cardiovascular disease clustered among those in their 40s and above, and 77% (10/13) of those aged 40 years above had abnormal SI values. Such individuals should pay particular attention to their health and follow medical advice. Although small in number, subjects with elevated SI values but no known cardiovascular disease should also remain alert for signs of emerging cardiovascular conditions.

### 4.3. Observation of HRV

Table 4 presents TP stratified by age and cardiovascular disease status. Based on the TP analysis, approximately 75% of subjects with TP values below 1500 ms^2^ were either CVD patients or individuals who reported fatigue at the time of testing. In addition, nearly 44% of CVD subjects had TP values below 1500 ms^2^. Therefore, in this study, a TP value of <1500 ms^2^ was defined as the threshold indicative of abnormal heart rhythm variability. The proportion of subjects with HRV abnormality was found to increase with age, mirroring the trends observed in SI and SIN. This demonstrates that older people may have a poorer physiological condition and physical performance compared to younger individuals, and the likelihood of unfavorable cardiovascular health increases with age. When further stratified by cardiovascular disease status, a higher proportion of subjects with abnormal TP was found in those aged 41 years and above with known cardiovascular disease, compared to those without the disease. This finding is in line with the hypothesis that HRV reflects cardiovascular risk, and the likelihood of an abnormally low TP value may be higher in individuals with known cardiovascular disease compared to those without.

Cross-analysis of medical charts and questionnaires showed that 69% of subjects with TP values below the normal range were diagnosed with cardiovascular disease. Among subjects with abnormal TP values but no known cardiovascular disease, 91% (10/11) self-reported fatigue from long hours of work under highly stressful conditions, being awake for more than 24 h, or chronic sleep deprivation. To avoid misclassification, subjects self-reporting fatigue due to external factors were excluded, and 96% (25/26) of subjects with abnormal TP values were found to have known cardiovascular disease. These results confirm that TP is an effective marker for evaluating cardiovascular risk.

### 4.4. Combination of the Three Indices

Table 5 lists the respective number of subjects stratified with abnormal SI values, SIN, or both. All subjects with abnormal SI values and SIN > 80% had known cardiovascular disease, whereas 70% of those with abnormal SI values and SIN > 40% had the disease. Therefore, the first set of thresholds effectively identified individuals with markedly impaired cardiovascular health, and these individuals should be classified as having a high risk of cardiovascular disease with poor cardiovascular conditions. A concurrent abnormal SI value and SIN >40% distinguished individuals without confirmed cardiovascular disease but who had impaired vascular elasticity and increased vascular stiffness; these individuals should begin monitoring their overall and cardiovascular states. This supports the effectiveness of the SI and SIN in assessing cardiovascular risk.

Cross-referencing the three cut-offs with medical charts and questionnaire responses revealed that the coexistence of elevated SI values, SIN > 40%, and TP < 1500 ms^2^ almost always indicated the presence of cardiovascular disease with declining cardiovascular performance, despite subjects being able to maintain normal daily function. By combining these three thresholds, we can assess cardiovascular disease risk while preventing false positive results in healthy individuals caused by transient issues, e.g., insomnia and fatigue. Among subjects with abnormal results, 80% of those showing at least two abnormal indicators had known cardiovascular disease.

## 5. Cardiovascular Health Status Assessment

Based on the age-related findings described in the previous sections, a six-tiered cardiovascular risk classification was proposed using three metrics (Table 6). The proportion of subjects with known cardiovascular disease within each tier is shown for reference. Almost 90% of subjects without known cardiovascular disease were found in Tiers 0–1, while 43.85% of subjects with known cardiovascular disease were in these tiers. This suggests that with appropriate management, individuals can maintain healthy and stable arterial performance, even in those with underlying cardiovascular disease. Subjects in Tiers 2–3 accounted for 50.87% of those with known cardiovascular disease, implying less favorable conditions characterized by elevated SIN values and reduced vascular elasticity compared to the general population. These individuals should stay vigilant and work to improve their diet and adherence to treatment. Notably, 10.44% of subjects without cardiovascular disease were also in these tiers; they should identify potential causes of impaired cardiovascular performance and make timely lifestyle, diet, and exercise modifications proactively. If their measurements do not improve after sustained lifestyle changes, medical evaluation is warranted. A Tier 4–5 classification reflects extremely poor cardiovascular health, with minimal normal waveforms and drastic effects on their physical performance and vitality. As shown in Table 6, no subjects without cardiovascular disease displayed such poor performance, with only a small proportion (5.26%) of subjects with cardiovascular disease classified in these high-risk categories. Individuals with such results should undergo immediate and comprehensive hospital examination and actively pursue interventions to improve their cardiovascular health, preventing the occurrence of acute and severe cardiovascular events.

The total study cohort of 191 subjects was further stratified by age using the six-tiered approach, as indicated in Table 6. Table 7 shows that at least 90% of subjects aged 1–40 years were in Tier 0, reflecting the expectation that younger individuals generally display good cardiovascular status. Of the few subjects aged 1–40 years not found in Tiers 0–1, the worst classification was Tier 2. This was due to zero cases of cardiovascular disease among all subjects aged 1–20 years, who were relatively young and healthy. Of the few subjects aged 21–40 years who had cardiovascular disease with limited symptoms, good adherence to medical management may have limited their risk to Tier 2. In contrast, only half of subjects aged 41–60 years had normal results (Tier 0), with more people appearing in Tiers 2–3, and sometimes even as high as Tier 4. Adding that only between 20 and 30% of subjects above 61 years were in Tier 0, these findings indicate deteriorating cardiovascular health becomes increasingly common with age. Elevated SIN ratios, reflecting worsening cardiovascular health, were particularly frequent in subjects aged 50 years and above, contributing to the higher proportion in Tiers 2–3 of these two age groups. Among subjects aged 50 years and above who were classified in Tiers 2–3, 71% (29/41) had known cardiovascular disease, while the remaining 29% (12/41) showed undetected cardiovascular conditions despite no recorded medical history. This underscores the need for closer monitoring of middle-aged and elderly individuals, in whom poor cardiovascular health may be prevalent. Although most younger subjects (1–40 years) demonstrated relatively good cardiovascular status, cases of abnormalities emerged, highlighting the importance of preventive measures starting from a young age.

In summary, this study analyzed PPG signals to assess alteration in vascular blood flow, and SI values were calculated to determine level of arterial stiffness. During development, it was observed that the presence of sine waveforms in PPG waves could lead to errors in SI estimation. To address this issue, SIN was introduced to represent the proportion of sine waveforms appearing in the entire PPG signal. SIN was further found to show significant association with both age and cardiovascular disease presence.

As arterial stiffness can result from various underlying factors, HRV was included as a supplementary indicator. A questionnaire was also administered to evaluate physical and psychological status to minimize confounding. Analyzing PPG signals from a total of 191 study subjects, SI values were found to significantly increase with age. Based on the normal SI reference range listed in Table 2, a higher proportion of subjects with known cardiovascular disease showed abnormal SI values compared to those without.

SIN ratios were significantly influenced by several factors. Subjects with SIN ratios greater than 80% had more than an 87% likelihood of being classified as having CVD. On the other hand, subjects with HRV below average (TP < 1500 ms^2^) were more likely to have known cardiovascular disease, whereas some subjects without known cardiovascular disease showed lower TP, likely due to sleep disturbance or physio-psychological fatigue.

Lastly, given the well-established association of obesity with cardiovascular disease, the study cohort was stratified by BMI. As shown in Table 8, the distribution of cardiovascular risk levels differed between overweight and normal-BMI subjects. According to the observation results, only 48.6% (17/35) of overweight subjects were classified as Tier 0 (normal), compared to 68.6% (107/156) of those with normal BMI, meaning that overweight individuals were less likely to exhibit good cardiovascular health. Of overweight subjects, 37.1% (13/35) had Tier 2–4 cardiovascular risk, noticeably higher than those with normal BMI (21.1%, 33/156). In particular, 5.7% (2/35) of overweight subjects were in Tier 4, compared to only 0.6% (1/156) of those with normal BMI, supporting that excess BMI increases the risk of extremely poor cardiovascular health.

These findings show that although it is an indirect indicator of cardiovascular health, BMI correlates with abnormalities in overall cardiovascular measurements. More specifically, subjects with excess BMI had a higher proportion of high cardiovascular risk compared to those with normal BMI. Notably, 49 (31.4%) of subjects with normal BMI also exhibited some level of cardiovascular risk, demonstrating that BMI alone can underestimate actual cardiovascular conditions. Thus, we do not recommend relying solely on BMI for screening, and a comprehensive assessment using multiple metrics, including those derived from PPG, offers a more accurate evaluation of cardiovascular conditions and allows for earlier risk detection.

## 6. Conclusions

This study proposes an easy-to-use device design for evaluating cardiovascular health status. By combining SI, SIN, and TP, stratifying measurements by age and known cardiovascular disease, and cross-checking with questionnaire results and medical charts, the present study demonstrates that a three-metric approach is highly effective in classifying cardiovascular health states and providing meaningful context. As an example, all subjects with concomitant SI values exceeding the age-specific normal range, SIN > 40%, and TP below average (1500 ms^2^) had known cardiovascular disease. Similarly, all subjects with SIN > 80% had cardiovascular disease. In addition, 80% of subjects with any two abnormal metrics—that is, with a risk level ≥ 3—had a prior diagnosis of cardiovascular disease. These results validate the robustness of the three-metric model for evaluating cardiovascular risk. Building on these findings, we propose a six-tiered cardiovascular health classification based on three simple PPG-derived metrics. This framework enables individuals to quickly self-evaluate cardiovascular status and take appropriate actions. The present study has successfully developed a constructive, simple, and rapid strategy for evaluating cardiovascular health using a non-invasive device, empowering the public to monitor their health, stay alert to potential risks, and seek timely medical attention to prevent serious or acute cardiovascular events.

Future work should involve more subjects for more reliable estimation thresholds, and conduct in-depth analyses according to biological factors such as sex and ethnicity to improve both the accuracy and global applicability of this approach, ultimately helping to establish a reliable warning system for cardiovascular health worldwide.

## Figures and Tables

**Table 1:** Proportion of participants with abnormal SIN ratios (SIN >40%) for identifying the presence of sine waveform by age group and cardiovascular disease status.

Age Group (Years)	CVD (n = 57)	NO CVD (n = 134)	Total
1~40 (88)	0% (0/1)	2.2% (2/87)	2% (2/88)
41~50 (23)	0% (0/9)	14.3% (2/14)	8.6% (2/23)
51~60 (32)	41.7% (5/12)	30% (6/20)	34.3% (11/32)
61~70 (28)	52.6% (10/19)	55.6% (5/9)	53.5% (15/28)
71~ (20)	66.7% (10/15)	60% (3/5)	65% (13/20)
Total average	43% (25/57)	13% (18/134)	22.5% (43/191)

**Table 2:** The upper and lower limits of the normal range of SI values.

Age	16~20	21~25	26~30	31~35	36~40	41~45	46~50	51~55
Lower limit	3.489	3.664	3.838	4.013	4.187	4.362	4.536	4.710
Upper limit	12.131	12.306	12.480	12.654	12.829	13.003	13.178	13.352
Age	56~60	61~65	66~70	71~75	76~80	81~85	86~90	91~95
Lower limit	4.885	5.059	5.234	5.408	5.583	5.757	5.931	6.106
Upper limit	13.527	13.701	13.875	14.050	14.224	14.399	14.573	14.748

**Table 3:** The percentage of participants with abnormal SI values, stratified by cardiovascular diseases and age groups.

Age Group (Years)	CVD (n = 57)	NO CVD (n = 134)	Total
1~20 (33)	-	6.1% (2/33)	6.1% (2/33)
21~40 (55)	0% (0/1)	7.4% (4/54)	7.3% (4/55)
41~60 (55)	18.2% (4/22)	3% (1/33)	9.1% (5/55)
61~ (48)	17.6% (6/34)	14.3% (2/14)	16.7% (8/48)
Total average	17.5% (10/57)	6.7% (9/134)	9.9% (19/191)

**Table 4:** Proportion of individuals in each age group with TP values below 1500 ms^2^.

Age Group (Years)	CVD (n = 57)	No CVD (n = 134)	Total
1~20 (33)	-	0% (0/33)	0% (0/33)
21~40 (55)	0% (0/1)	1.9% (1/54)	1.8% (1/55)
41~60 (55)	40.9% (9/22)	21.2% (7/33)	29.1% (16/55)
61~ (48)	47% (16/34)	21.4% (3/14)	39.5% (19/48)

**Table 5:** Percentages of participants with abnormal SI values, SIN ratios, and TP stratified by presence or absence of cardiovascular disease.

	CVD (n = 57)	No CVD (n = 134)
SIN > 40%	43.9% (25/57)	13.4% (18/134)
SIN > 80%	10.5% (6/57)	0.7% (1/134)
SI abnormal and SIN > 40%	12.28% (7/57)	2.2% (3/134)
SI abnormal and SIN > 80%	1.75% (1/57)	0 (0/134)
SI abnormal and SIN > 40% and TP < 1500 ms^2^	3.5% (2/57)	0 (0/134)
SI abnormal and SIN > 80% and TP < 1500 ms^2^	0% (0/57)	0% (0/134)

**Table 6:** Proportion of subjects for each risk value.

Level	CVD (n = 57)	No CVD (n = 134)
0	35.08% (20/57)	77.6% (104/134)
1	8.77% (5/57)	11.94% (16/134)
2	38.59% (22/57)	8.95% (12/134)
3	12.28% (7/57)	1.49% (2/134)
4	5.26% (3/57)	0% (0/134)
5	0% (0/57)	0% (0/134)

**Table 7:** Proportion of each age group accounting for each risk value.

Age Group (Years)	Level 0	Level 1	Level 2	Level 3	Level 4
1~20 (33)	90.9% (30/33)	9% (3/33)	0% (0/33)	0% (0/33)	0% (0/33)
21~40 (55)	90.9% (50/55)	5.4% (3/55)	3.6% (2/55)	0% (0/55)	0% (0/55)
41~60 (55)	56.3% (31/55)	14.5% (8/55)	20% (11/55)	7.2 (4/55)	1.8% (1/55)
61~ (48)	27% (13/48)	14.6% (7/48)	43.7% (21/48)	10.4% (5/48)	4.16% (2/48)

**Table 8:** Proportion of each BMI group accounting for each risk value.

Level	BMI Overweight (n = 35)	BMI Normal (n = 156)	Total (n = 191)
0	48.6% (17/35)	68.6% (107/156)	64.9% (124/191)
1	14.3% (5/35)	10.3% (16/156)	11% (21/191)
2	20% (7/35)	17.3% (27/156)	17.8% (34/191)
3	11.4% (4/35)	3.2% (5/156)	4.7% (9/191)
4	5.7% (2/35)	0.6% (1/156)	1.6% (3/191)
5	0%	0%	0%

## Data Availability

The data supporting the findings of this study are available from the corresponding author upon reasonable request, due to privacy restrictions.

## Abbreviations

The following abbreviations are used in this manuscript:

PPG	Photoplethysmography
HRV	Heart Rate Variability
SI	Stiffness Index
SIN	Sine Wave Ratio
